# Primary resection versus neoadjuvant chemoradiation followed by resection for locally resectable or potentially resectable pancreatic carcinoma without distant metastasis. A multi-centre prospectively randomised phase II-study of the Interdisciplinary Working Group Gastrointestinal Tumours (AIO, ARO, and CAO)

**DOI:** 10.1186/1471-2407-7-41

**Published:** 2007-03-06

**Authors:** Thomas B Brunner, Gerhard G Grabenbauer, Thomas Meyer, Henriette Golcher, Rolf Sauer, Werner Hohenberger

**Affiliations:** 1 Department of Radiation Oncology of the University at Erlangen-Nuremberg, Erlangen, Germany; 2 Department of Surgery of the University at Erlangen-Nuremberg, Erlangen, Germany

## Abstract

**Background:**

The disappointing results of surgical therapy alone of ductal pancreatic cancer can only be improved using multimodal approaches. In contrast to adjuvant therapy, neoadjuvant chemoradiation is able to facilitate resectability with free margins and to lower lymphatic spread. Another advantage is better tolerability which consecutively allows applying multimodal treatment in a higher number of patients. Furthermore, the synopsis of the overall survival results of neoadjuvant trials suggests a higher rate compared to adjuvant trials.

**Methods/Design:**

As there are no prospectively randomised studies for neoadjuvant therapy, the Interdisciplinary Study Group of Gastrointestinal Tumours of the German Cancer Aid has started such a trial. The study investigates the effect of neoadjuvant chemoradiation in locally resectable or probably resectable cancer of the pancreatic head without distant metastasis on median overall survival time compared to primary surgery. Adjuvant chemotherapy is integrated into both arms.

**Discussion:**

The protocol of the study is presented in condensed form after an introducing survey on adjuvant and neoadjuvant therapy in pancreatic cancer.

## Background

Ductal carcinoma of the head of the pancreas represents a considerable problem in oncology with a mortality rate of 8–12/100,000 and per year. Pancreatic carcinoma is the fifth leading cause of cancer death in Germany. For all stages, 5 year overall survival rate is 6% form en and 3% for women[1]. Curative (R0-) resection is only possible in 20% of all patients but even then the 5 year overall survival rate is only 25% – even in cancer centres[2]. Median overall survival time of 18–25 months after R0-resect5ion reflects the bad prognosis of this disease[3].

### Surgical therapy

Patients with pancreatic carcinoma who are presented in surgical departments have tumours that are unresectable in 70–80% because of locally advanced disease or distant metastases. Surgery results in about 25% in a palliative situation (R1-/R2-resection). These patients have no chance of cure (Tumour Registry of the University Hospitals of Erlangen and [3]).

The bad prognosis for patients with pancreatic cancer is caused above all by:

- Early invasion of the mesenteric root and of the central vessels (superior mesenteric artery and superior mesenteric vein, portal vein). In spite of resection of infiltrated vessels by e.g. tangential or segmental portal vein resection and successive reconstruction, almost all of these patients will eventually die within a year.

- Frequent and extensive lymphatic metastases in up to 75%. Between 1983 and 1997, 647 patients with ductal adenocarcinoma of were registered at the registry of the surgical department of the University of Erlangen. Survival for patients with stage III (T1-3; N+; UICC 1997) was 3% without adjuvant therapy after 5 years.

This scenario emphasizes that surgical therapy alone is not sufficient for pancreatic carcinoma and that prospective investigation of additional modalities is crucial. The high frequency of local relapse and of distant metastasis is the main reason for tumour progression. The rate of local relapse without adjuvant therapy is about 80% [4-6] and can be reduced to rates up to 26% after adjuvant chemoradiation (CRT) [5,7-9].

The rationale of adjuvant therapy is to reduce the rate of local relapse and distant metastasis. Neoadjuvant CRT can additionally increase the rate of resections with clear margins (R0-resection).

### Actual assessment of adjuvant therapy

The results of three randomised studies of adjuvant therapy are summarised in table 1[10-12]. In the GITSG trial the patients were randomised either for 5-fluorouracil (5-FU) based CRT and sequential weekly 5-FU maintenance 5-FU chemotherapy or for observation[10]. The patients in the treatment arm had longer survival times compared to patients with resection only (20 months vs. 11 months). The EORTC randomised in a following larger phase III study 218 patients with adenocarcinoma of the pancreas or of the peri-ampullary region either for CRT or for no further treatment after Whipple's procedure. [11]. In a subgroup analysis the median survival time for patients with pancreatic carcinoma was 17.1 months for patients with CRT compared to only 12.6 months after surgery alone (p = 0.099). Although, the prolongation of survival after adjuvant therapy did not reach statistic significance the EORTC trial confirmed that the survival of patients without adjuvant therapy is poor (10–12 months).

The ESPAC-1 trial [12,13] included 541 patients and 289 of them were randomised for a 2 × 2 factorial study design that addressed the patients to the study arms observation, chemotherapy, CRT or CRT followed by chemotherapy. CRT consisted in bolus 5-FU and radiotherapy of 40 Gy in a split course technique. Chemotherapy was given as six cycles of bolus 5-FU (425 mg/m^2^) and leukovorin (20 mg/m^2^) per day for 5 days with repetition after 28 days. Recently, the results of the ESPAC-1 trial have been reported with restriction on the patients that had been randomised to the 2 × 2 factorial study design. [12]. This analysis showed lower survival for patients after CRT compared to the patients without CRT (15.9 months vs. 17.9 months). Vice versa, patients with chemotherapy had longer survival in comparison with those who had no chemotherapy (20.1 months vs. 15.5. months; p = 0.009). These results question the role of CRT as a component of adjuvant therapy. The interpretation of the results of the ESPAC-1 study is very difficult for a number of different reasons (cf. the editorial „One lesson to be learned from this well-intentioned study is that a straightforward analysis driven by the factorial structure is not always as simple as it seems“ [14]). Remarkably, the patients of the best arm of the ESPAC-1 trial have a median survival time which is identical with the patients form the GITSG trial that have been achieved two decades earlier.

At the 2005 annual meeting of the American Society of Clinical Oncology (ASCO) the preliminary results of another phase III trial (CONKO-001) of adjuvant therapy has been presented [15,16]. Within six weeks after resection 386 patients (N+ in 72% and R1 in 18%) were randomised to gemcitabine (179 patients) or observation (177 patients); 12 patients were excluded. Gemcitabine was given with 1000 mg/m^2 ^on days 1, 8 and 15 every 4 weeks for 6 months. The primary endpoint was disease free survival (DFS) and the secondary endpoints overall survival and toxicity. At the time of analysis 68% of the patients had progress. Median DFS was significantly different (p < 0.05): after gemcitabine 14.2 months vs. 7.5 months without. Data were not yet ready for analysis of overall survival. The final results are expected for late 2006.

More knowledge about adjuvant therapy of pancreatic carcinoma are expected from the currently active studies: EORTC 40013/22012 (gemcitabine vs. gemcitabine + CRT + gemcitabine), ESPAC-3 (5-FU vs. gemcitabine), RTOG (5-FU + CRT + 5-FU vs. Gem + RCT + Gem), CapRI-study (5-FU vs. CRT with 5-FU/cDDP/IFN-α) [17].

The current situation on adjuvant therapy can be summarized with three aspects: (1) the general impression of randomised studies is that an improvement of the survival rates of about 10% is realistic. (2) The suitability of DFS as an endpoint for adjuvant trials is very restricted. Ultrasound for follow up (as used in the CONKO-1-study in week 8. and 16. after surgery) is not able to detect local recurrence after the abdominal anatomic changes of Whipple's procedure. Overall survival remains the most robust endpoint for adjuvant studies. (3) The following questions remain to be answered by further trials: the type of adjuvant chemotherapy (5-FU vs. gemcitabine), the role of adjuvant chemoradiation, performed with modern standards, the question if patients with R1-resections necessitate different therapy than patients with R0-resections, and the evaluation of the value of neoadjuvant chemoradiation.

### Neoadjuvant therapy of pancreatic carcinoma

Neoadjuvant therapy of pancreatic carcinoma is able to increase the resectability rate with clear margins (R0-resection)[18,19] and to decrease the rate of local relapse[20]. Additionally, neoadjuvant trials report a decrease of the rate of metastastic lymph nodes (30–48%)[20,21] compared to initial surgery (60–80%) [2,22-26]. Both effects improve the prognosis of patients with pancreatic carcinoma. Furthermore, neoadjuvant therapy is better tolerated than adjuvant therapy resulting in a higher number of patients who will tolerate the intended therapy (reviewed in [27]): 20% of the initially treated patients are unresectable intra-operatively. Another 25–50% of the patients do not receive adjuvant therapy because of insufficient recovery from surgery or because of patient refusal of therapy. In total, only 40–60% of primary operated patients eventually will be administered the intended therapy. In contrast, complete therapy (neoadjuvant CRT and resection) can be given in 60–65% of the patients with tumours estimated to be resectable at diagnosis. Rapid disease progression is the reason why 20–25% will not be resected after neoadjuvant CRT. These patients experience rapid progression because of aggressive tumour biology not only during or after neoadjuvant CRT but also after primary resection. In a neoadjuvant concept they will be spared futile morbidity of Whipple's procedure. Another 20% of the patients are not resected intra-operatively because of extrapancreatic disease. For the latter patients optimal therapy for locally advanced pancreatic carcinoma has already been given which is chemoradiation

To date, there is no prospectively randomised phase III-study for neoadjuvant therapy. Table 2 shows a summary of the largest published studies. Most of these series are single institutional ([20,21,28,29]). The largest published series is from the M.D. Anderson Cancer Centre and reports on 132 patients who have been treated between 1990 through 1999. A remarkable 88% rate of R0-resections is well above the results after initial resection. Median overall survival time of 21 months is excellent in the light of a 43% rate of vascular resections. After CRT patients with nodal spread had worse prognosis than those without nodal spread as well as patients with male gender in multivariate analysis. The first site of relapse was exclusively local in 5% of the patients. Another 5% of the patients had combined local and distal relapse. The rate of perioperative lethality was not increased (2/132). Furthermore, 4 treatment related deaths occurred as a consequence of gastrointestinal bleedings (3 patients) and after thrombosis of a reconstructed portal vein (1 patient). Good response of the tumours at tumour regression grading correlated with a trend to longer survival.

The correlation between good response and survival was significant in the Duke University series (1994 and 2004) where 70/193 patients were resected after CRT. Patients with good response in tumour regression grading had a significant prolongation of survival. Furthermore, the histological description of tumour necrosis was a bad prognostic factor. The median overall survival of 39 months of patients with primary unresectable tumours who underwent resection after CRT is remarkable. As in the Houston series[20] patients without nodal metastasis had longer survival. The relative low rate of nodal disease (30%) in comparison with initial resection[2,22-26] allows to deduct sterilisation of nodes with disease after CRT. It is also important to acknowledge that resection could be performed after CRT in 18% of the patients who had initially unresectable tumours. In a trial published by Wilkowski et al. this rate was even higher (14/33 patients = 42%)[30]. This means that resectability needs to be re-evaluated after CRT.

Median survival of neoadjuvantly treated (n = 61) vs. adjuvantly treated patients (n = 55) was longer for neoadjuvantly treated patients in a study from Philadelphia which compared both approaches (median OS = 23 vs. 16 months)[28]. Positive margins and lymphatic disease had a significant influence on survival. Another study compared primary surgery (n = 91) of patients with initially resectable tumours with neoadjuvant CRT of patients with locally advanced tumours (n = 68). Survival was longer for patients after CRT with and without subsequent resection compared to patients with primarily resected tumours (23.6 vs. 14 months of mOS). Median overall survival was 32 months for 20/68 patients with resection after CRT. Curative resection (R0) could be increased by 11% as a consequence of neoadjuvant CRT.

In summary, the value of neoadjuvant CRT can be described by four short statements:

(1) there are several safe and sufficiently tested schedules for neoadjuvant CRT. However, gemcitabine containing schedules probably can achieve a higher rate of pathologic response compared to 5-FU based protocols[31]. Simultaneous CRT with gemcitabine and cisplatin is able to produce even in locally advanced pancreatic carcinoma good local response with a high rate of secondary respectability with good tolerability[30,32,33].

(2) The survival rates of the synopsis of neoadjuvant trials are longer in comparison with the survival rates after resection and adjuvant therapy.

(3) The accurate definition of resectability is a prerequisite for the quality of neoadjuvant studies. Resectability needs to be based upon pretherapeutic imaging which is preferentially performed by helical CT scanning and the description of the exact extent of vascular involvement [34-36].

(4) Even if pretherapeutic histological proof is not always possible it can be achieved in a high percentage when diagnostic and therapeutic disciplines interact closely[37].

## Methods/Design

This trial aims to answer the question if neoadjuvant CRT with gemcitabine and cisplatin can prolong overall survival of patients with ductal adenocarcinoma of the pancreatic head in comparison with primary resected patients.

**Figure 1:**
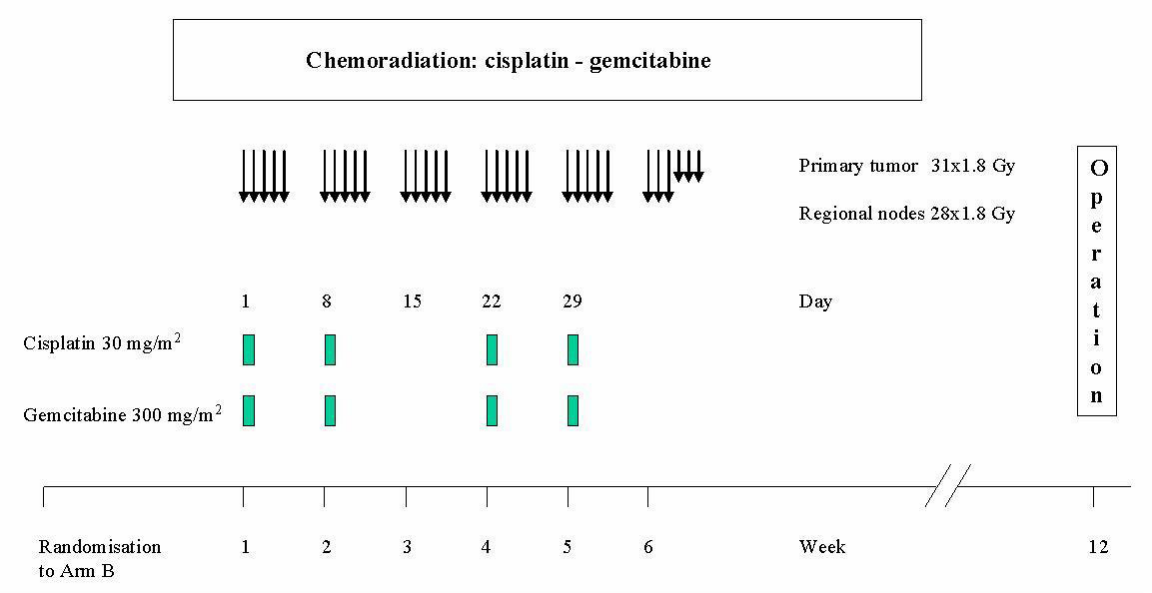
Treatment schedule.

The treatment algorithm (Fig. 1) depicts the treatment flow. Tables 3 and 4 lists all inclusion and exclusion criteria. It is mandatory for inclusion to obtain the histological proof of diagnosis e.g. via CT guided biopsy or conclusive brush cytology, and to perform a multi slice CT scan of the abdomen showing a resectable or probably resectable tumour [34]. If informed consent has been obtained prior to surgery, randomization can also be performed intra-operatively during laparoscopy

**Arm A **consists of partial (subtotal) duodenopancreatectomy with defined lymphatic dissection followed by adjuvant chemotherapy.

**Arm B **consists of neoadjuvant CRT (Fig. 2), followed by tumour resection and then by adjuvant chemotherapy.

### Chemoradiation

Conventional fractionated, 3-d-conformal irradiation is administered with a total dose of 55.8 Gy (tumour region) or 50.4 Gy (regional lymph nodes) respectively[38]. Simultaneously gemcitabine (300 mg/m^2^) and 30–60 minutes later cisplatin (30 mg/m^2^) or administered on days 1, 8, 22 and 29 both as a 30 minute IV infusion. Six weeks after completion of CRT, a restaging CT scan is scheduled to exclude tumour progress. Thereafter, surgery is performed.

### Surgical therapy

Partial duodenopancreatectomy is done if the tumour appears to be resectable after laparotomy. The minimal requirements for surgery are:

• Transsection of the hepatocholedochal duct slightly distally to the junction of the cystic and the hepatic duct.

• Transsection of the pancreas minimally at the level of the left edge of the portal vein.

The resection plane of the specimen should be analysed histologically if possible already at the intraoperative fresh frozen section. However, it is mandatory to perform histological analysis of the postoperatively paraffin embedded tissue to exclude positive margins (R1-resection).

In both arms, adjuvant chemotherapy for 6 months according to the protocol of the CONKO-001 study (Neuhaus et al. 2006 JAMA in press) is recommended.

### Quality of life

Evaluation of quality of life is done with a standardized questionnaire (EORTC QLQ-C30 with additional module).

### Follow up

Follow up examinations are planned every 3 months fort he first 2 years after surgery and thereafter every 6 months for at least 3 years. Examinations consist of a physical examination, monitoring of the course of CA-19-9 and ultrasound of the abdomen. Staging is complemented by CT abdomen and a chest X-ray every 6 months if local or distant failure is suspected.

## Discussion

The trial is carried out as a multi-centre trial with centres from Germany, Switzerland and Austria. The participating centres offer all techniques of 3-d-conformal irradiation. They all perform more than 15 pancreaticoduodenectomies per year, each. Extensive experience in combined multimodality treatment approaches is provided. Therefore, all essential requirements to conduct the present study are met at the participating departments. With the present trial, we aim at improving outcome in patients with resectable pancreatic adenocarcinoma by adding neoadjuvant chemoradiation to the current treatment standard consisting of resection followed adjuvant chemotherapy.

## List of abbreviations

CRT = chemoradiation

GITSG = Gastrointestinal Tumour Study Group

5-FU = 5-fluorouracil

EORTC = European Organisation for Research and Treatment of Cancer

**ESPAC-1 **= European Study Group for Pancreatic Cancer

CONKO-001 = Charité Onkologie Clinical. Studies in GI Cancer

DFS = disease-free survival

RTOG = Radiation Therapy Oncology Group

Gy = Gray

## Competing interests

The author(s) declare that they have no competing interests.

## Authors' contributions

T.B.B. wrote the manuscript and is the study coordinator for the neoadjuvant component of the trial

G.G.G. was involved in the study design, assisted in writing the manuscript and is the principal investigator for chemoradiation within the trial

T.M. is the study coordinator for the surgical component of the trial

H.G. is the main responsible physician of the surgical study office and supervises correct documentation of the trial

R.S. participated in the design of the study and helped to support the trial among the ARO Society (German Working Group of Radiation Oncology)

W.H. contributed to the study design and he is the principal surgical investigator of the trial. All authors read and approved the final manuscript.

## Pre-publication history

The pre-publication history for this paper can be accessed here:



## Figures and Tables

**Table 1:** Phase III studies of adjuvant 5-fluorouracil based therapy#.

Study year	Number of patients	Inclusion criteria Resection status	5-FU based therapy (months)	Follow-up (months)	p	Preoperative diagnostics	Postoperative Imaging	Inked margin
GITSG-1985 [10]	49	R0	RCT 21.0	10.9	0.005	No	No	No
EORTC-1999 [11]	114*	R0	RCT 17.1	12.6	0.099	No	No	No
ESPAC-1-2004 [12]	289§	R0 or R1	Cx 21.6	16.9	n.a.	No	No	No

# Median survival rates from three randomised studies in patients with resected pancreatic carcinoma. None of these trials used either high quality preoperative imaging to evaluate resectability, or postoperative imaging, to exclude tumour persistence or distant metastasis.

* The EORTC trial comprised 218 patients with periampullary and pancreatic carcinoma. The results shown here are based upon the 114 patients with pancreatic carcinoma.

§ 541 patients were included into ESPAC-1, but only 289 were included into 2 × 2 factorial randomisation. Trial arms: observation, chemotherapy, chemoradiation, chemoradiation followed by chemotherapy. The table gives the survival rates fort he best treatment arm (chemotherapy) and observation.

**Table 2:** Selected studies of neoadjuvant chemoradiation.

**Study Institution**	**Number of patients**	**Total dose of XRT (Gy)**	**Chemo-therapy (mg/m**^**2**^**)**	**Median OS (months)**	**1(2,4,5)-year-OS-rate (%)**	**Rate local recurrence (%)**	**Rate of resectability (%)**	**Rate of clear resections**
***Hoffman et al. 1998 ***[39, 40] multi-centric	53	50.4	5-FU/MMC	9.7 all pts 15.7 res	*2y: *27 res	24/53 (26 %) 3/24 (13%) res	24/53 (45%)	n.a
***Snady et al. 2000 [29] ***Mount Sinai	159 68* (20 res^§^) 91 adj	54 + 14 Gy	5-FU/cDDP/Streptozotocin	23.6* (32 res) 14.0	*1 y: *86*(89)vs.64 *2y: *58*(60) vs.32 *3y: *27*(40)vs.17	n.a.	20/68 (29%) -	95% neo 84% adj
***Breslin et al. 2001 [20] *** *MDACC*	132	45 or 50.4 Gy or 10 × 3 Gy ± IORT	5-FU, or Tax or Gem	21	*1y: *75 *2y: *40 *5y: *23	8/132 (6%)	-	88%
***Sasson AR et al.2003 [28] ***FCCC	116 61 neo 55 adj	50.4	5-FU/MMC or Gem	all 18 neo 23 adj 16	n.a.	n.a.	-	39% n.a. n.a.
***White et al. 2005 [21] ***Duke	193 i.p.r.:102 i.l.f.: 91	45 + 5.4 Gy	5-FU or Gem	23 39 i.p.r. 20 i.l.f.	*3y: *37 res *5y: *27 res	n.a.	70/193 (36%) 54/102 (53%) 16/91 (18%)	73% n.a. n.a.

Abbreviations: 5-FU = 5-fluorouracil; adj = adjuvant therapy; cDDP = cisplatin; FCCC = Fox Chase Cancer Center, Philadelphia, PA; Gem = gemcitabine; Gy = Dose in Gray; i.p.r = initially potentially resectable; i.l.f. = initially locally advanced, MDACC = M.D. Anderson Cancer Center Houston, TX; MMC = mitomycin C; n.a. = not available; neo = neoadjuvant; OS = overall survival; res = resected patients, RT = radiotherapy; Tax = paclitaxel; vs. = versus. *initially unresectable patients ± resection after chemoradiation; ^§ ^this study indicates overall survival as explained: (1) patients with chemoradiation (2) numbers in brackets: patients with chemoradiation and resection (3) right to 'vs.': patients after primary resection; ^§ ^numbers in brackets in this row give results of the 20/68 resected patients with RCT.

**Table 3:** Inclusion criteria

• Histologically proven **ductal adenocarcinoma of the pancreatic head **(tumours of the pancreatic head right to the left edge of the superior mesenteric vein including the uncinate process), stage I- IV A according to UICC 1997. Histological proof should be performed via percutaneous CT- or ultrasound guided biopsy (up to 3 attempts) if impossible by endoscopic biopsy or endoscopic cytology (brush cytology with tumour cells).
• **No infiltration of extrapancreatic organs **with the exception of the duodenum
• pancreatic tumour as imaged in **multi slice spiral CT (slice collimation 3 mm) **estimated to be resectable or probably resectable by an experienced surgeon on the base of the following criteria: vascular involvement ≤ 180° of one of the peripancreatic major vessels (portal vein, confluent of superior mesenteric vein and, splenic vein, superior mesenteric artery, celiac trunk with its major branches splenic artery and hepatic artery, superior mesenteric vein) (Lu et al., 1997).
• maximally **1 enlarged (> 1 cm) regional lymph node **in thin slice spiral CT, without signs of vessel infiltration
• no distant metastasis (in X-ray chest, abdominal CT and laparoscopy)
• no peritoneal spread (laparoscopy prior to randomization is recommended)
• age at treatment initiation **minimally 18 years**
• **Karnofsky performance status ≥ 70 **(70 – not capable to work, independent)
• Written informed consent of the patient

**Table 4:** Exclusion criteria

• ampullary carcinoma (tumours origination from the ampulla, the papilla or at the junction of the ampulla and the papilla)
• carcinoma of the pancreatic corpus or tail (tumours between the left edge of the superior mesenteric vine and the left edge of the aorta respectively between the left edge of the aorta and the splenic hilum)
• Non-ductal adenocarcinoma of the pancreas (e.g. cystadenocarcinoma, neuroendocrine tumours, etc.)
• Tumour specific prior treatment
• Recurrent tumour
• Peritoneal spread
• distant metastasis
• 2 or more enlarged lymph nodes (> 1 cm), with suspicion of metastatic spread based upon morphology in CT scan.
• Infiltration of extrapancreatic organs except of the duodenum
• vascular involvement > 180° of minimally one of the peripancreatic major vessels (portal vein, confluent of V. mesenterica sup. and V. lienalis, A. mesenterica superior, coeliac trunk, V. mesenterica superior), stenosis or occlusion of one of the mentioned vessels. Resectability only if vascular resection is performed (including portal vein and superior mesenteric vein).
• Prior or synchronous malign neoplasia (Except: non-melanomatous skin cancer and curatively treated carcinoma in situ of the uterine cervix and tumour treated by surgery alone with 10 years of disease-free survival)
• Participation at a clinical trial within the last three months prior to inclusion
• Hepatic cirrhosis with platelets < 100 000/mm3 or PTT < 70%
• Serum creatinine > 1.5 mg/dl, creatinine-clearance < 70 ml/min (24 h collection of urine; because of planned chemotherapy with cisplatin)
• Severe cardio-pulmonary concomitant disease (cardiac insufficiency NYHA III/IV, arrhythmia Lown III/IV, pathologic findings at cardiac ultrasound (pathologic ejection fraction), respiratory global insufficiency, or any other severe disease that could interfere with complete therapy as rated by the surgeons or radiation oncologists who participate in the treatment
• HIV-infection
• pregnancy or insufficient contraception
• Age < 18 years
• Karnofsky performance status < 70
• doubtful understanding or contractual capacity of the patient
